# Erythropoietin regulates osteoclast formation via up-regulating PPARγ expression

**DOI:** 10.1186/s10020-024-00931-7

**Published:** 2024-09-15

**Authors:** Xiao Liu, Mengxue Zhou, Yifan Wu, Xiang Gao, Lei Zhai, Liang Liu, Huan Geng

**Affiliations:** 1 Department of Orthopedics, The Second Affiliated Hospital, School of Medicine, Zhejiang University, Hangzhou, 310058 China; 2 Key Laboratory of Tea Biology and Resource Utilization of Ministry of Agriculture, Tea Research Institute, Chinese Academy of Agricultural Sciences, Hangzhou, 310008 China; 3 Meiao Dingcheng Clinic Limited Company, Tianjin, 300000 China

**Keywords:** EPO, Bone remodeling, Trabecular, Osteoblast differentiation, Osteocyte

## Abstract

Erythropoietin (EPO), expressed in red blood progenitor cells, primarily regulates erythropoiesis by binding to its receptor. Besides anemia, recent studies have identified new therapeutic indications for EPO that are not connected to red blood cell formation. Elevated EPO levels harm bone homeostasis in adult organisms and are associated with increased osteoclast; however, the underlying molecular mechanisms remain unclear. This study demonstrated that EPO enhanced osteoclast differentiation and bone resorption in vitro. We showed that EPO promoted osteoclast formation by up-regulating PPARγ expression through activating the Jak2/ERK signaling pathway. Consistently, PPARγ antagonists rescued the hyperactivation of osteoclasts due to EPO, while PPARγ agonists reversed the EMP9-mediated decrease in osteoclast differentiation. Further, exposing female mice to EPO for two months led to a decrease in bone mass and increased osteoclast numbers. The present results suggested that EPO promotes osteoclastogenesis by regulating the Jak2/ERK/ PPARγ signaling pathway. From a clinical perspective, the risk of compromised bone health should be considered when using EPO to treat anemia in post-operative patients with intertrochanteric fractures of the femur, as it could significantly impact the patient’s recovery and quality of life.

**Supplementary Information:**

The online version contains supplementary material available at 10.1186/s10020-024-00931-7.

## Introduction

Erythropoietin (EPO) is a renal hormone the kidney produces that effectively promotes erythropoiesis. Clinically, recombinant EPO is used to treat anemia caused by chronic kidney disease, cancer, or chronic blood loss (Kaneko et al. 2022). Besides hematopoietic tissue, EPO can stimulate non-erythrocyte response mediated by the erythropoietin receptor (EPOR), which is also expressed in other normal tissues, such as the brain and bone. Extensive research has been conducted over the past two decades to examine the impact of EPO on bone metabolism (Suresh et al. 2020a, b, c). While many studies have shown that exogenous administration of high doses of EPO induces bone loss in adult mice (Singbrant et al. 2011; Shiozawa et al. 2010; Rauner et al. 2016), there is limited data supporting a direct impact of EPO on bone metabolism. Therefore, the exact role of EPO in bone remodeling and its mechanism of action remains unclear.

The maintenance of bone mass depends on the dynamic balance between osteoblast-mediated bone formation and bone resorption by osteoclast-mediated bone resorption (Han et al. 2020). Osteoclasts are the primary, if not the only, cells responsible for bone resorption (Wu et al. 2017). These large multinucleated cells differentiate from monocyte/macrophage cell lines stimulated by macrophage colony-stimulating factor (M-CSF) and receptor activator of NF-κB ligand (RANKL) (Murata et al. 2022). RANKL, expressed in osteocytes, bone marrow stromal cells and activated T cells, is a central positive regulator of osteoclast formation (Tsukasaki et al. 2020). When RANKL binds to its receptors, RANK, it activates several intracellular signaling pathways, such as NF-κB and MAPK-AP-1 signaling pathways. Activation of these signaling pathways converges to stimulate the expression and activates the function of transcription factors to drive the osteoclast differentiation program (Tsukasaki et al. 2020). Recently, Suresh et al. demonstrated that pre-osteoclasts express functional EPOR, and they found that mice with overexpressed EPO had reduced bone mass due to an increased number of osteoclasts. This suggests that EPO may reduce bone mass by directly promoting osteoclast differentiation. Nevertheless, the mechanisms by which EPO regulates osteoclast differentiation remain unknown.

In this study, we examined the effectiveness of EPO on the differentiation of osteoclasts induced by RANKL and osteoclastic bone resorption. Our results demonstrated that EPO could promote osteoclastogenesis. The molecular mechanisms underlying EPO’s ability to promote osteoclast differentiation involve increased intracellular PPARγ expression. Furthermore, EPO increases PPARγ expression primarily by inactivating Jak2/ERK.

## Materials and methods

### Cell culture and differentiation into osteoclasts

Mature osteoclasts were generated from bone marrow macrophages (BMMs) as described previously (Geng et al. 2017). Briefly, primary bone marrow cells were isolated from the femora of 5-week-old C57BL/6 mice. We cultured primary bone marrow cells in the complete medium containing 50 ng/mL recombinant M-CSF (R&D Systems, Minneapolis, MN, USA) for 3 days. Adherent cells at this stage were considered M-CSF–dependent BMMs and used as osteoclast precursors (cells at day 0). For osteoclastogenesis, the resultant BMMs were plated in 48-well plates and cultured in osteoclastogenic medium that consisted of 30 ng/mL M-CSF and 50 ng/mL RANKL for 4 days. We identified osteoclasts by staining for tartrate-resistant acid phosphatase (TRAP) activity. The cells were fixed with 4% paraformaldehyde and stained using the TRAP staining kit (Sigma-Aldrich, 387 A-1KT) based on the instructions received from the manufacturers. The TRAP^+^ multinucleated (> 3 nuclei) cells were counted as osteoclasts. According to the experimental requirements, 20IU/ml EPO (Sunshine Pharmaceutical, Shenyang, China), 0.5 mg/ml EMP9 (MedChemExpress, Wuhan, China), 20 µM TZD (Sigma, St. Louis, MO, USA), or 10 µM GW9662 (Sigma, St. Louis, MO, USA) were added into the osteoclast differentiation process.

### Immunofluorescence staining

The cells were fixed in 4% formaldehyde for 15 min, then the membranes were broken with 0.1% Triton X-100 for 10 min. The cells were treated with a blocking buffer and then exposed to primary antibodies targetting EPOR or PPARγ at 4 °C overnight. Subsequently, the cells were incubated with secondary antibody-labeled fluorescent at room temperature for 1 h. The cell nuclei were stained with DAPI, and fluorescence images were acquired using confocal laser scanning microscopy (Smartproof 5, Carl Zeiss, Oberkochen, Germany).

### Western blotting

The cells were washed twice with PBS and lysed in an ice-cold RIPA Lysis Buffer (Beyotime, China) containing protease inhibitors (Beyotime, China) for 20 min to extract total protein. Then, the supernatant was collected and centrifuged for 10 min at 12,000 g and 4 °C to obtain the protein sample. An equal amount of protein (50 µg) was mixed with sodium dodecyl sulfate-polyacrylamide gel electrophoresis (SDS-PAGE) loading buffer (Beyotime, China) and then heated at 95 °C for 5 min. Subsequently, the protein sample was separated using SDS-PAGE on 14% polyacrylamide gels and then transferred to polyvinylidene difluoride membranes. The membranes were blocked with 5% skim milk and then examined using specific primary antibodies against EPOR (ABclona; Catalog: A2917), phospho- (p-)Jak2, Jak2, p-ERK, ERK, GAPDH and PPARγ overnight at 4 °C, followed by incubation with corresponding secondary antibodies at room temperature for 1 h. Finally, immunoreactive bands were detected with ECL reagents. All antibodies were purchased from ABclona.

### Actin ring formation assay

BMMs were seeded in confocal dishes and incubated in a complete inducing medium containing M-CSF and RANKL for four days to form actin rings. Subsequently, the cells were washed twice with PBS and fixed in 3.7% paraformaldehyde for 15 min. The cells were washed with PBS three times. Then, the cells were stained with rhodamine-conjugated phalloidin (Cytoskeleton, Inc., Denver, CO, USA), while the cell nucleus was stained with DAPI (Sigma–Aldrich) for 10 min. The cells were photographed using a confocal laser scanning microscopy.

### Bone resorption assay

BMMs (2 × 10^4^ cells/well) were cultured in Osteo Assay Surface 24-well plates (Corning, NY, USA) coated with a calcium phosphate substrate. BMMs incubated in complete inducing medium containing M-CSF and RANKL for 6 days. Subsequently, the cells were washed with 10% sodium hypochlorite and rinsed with water three times. The images were captured using an ordinary light microscope. The percentage of the resorbed bone surface area was quantified using ImageJ software.

### RT-qPCR

The total RNA was extracted using TRIzol according to the instructions received from the manufacturer. Then, RT-qPCR was performed to evaluate the transcription of osteoclast-related genes, including *Acp5*, *Ctsk*, *Mmp9*, *Calcr*, *NFATc1*, and *c-fos* using a SYBR Green qPCR Master Mix Kit (Applied Biosystems, Foster City, CA, USA) according to the manufacturer’s recommendations. The relative mRNA expression was normalized with GAPDH. The primers used in this experiment was as follows:

*Acp5*, forward 5′-CTGGAG TGCACGATGCCAGCGACA-3′ and reverse 5′-TCCGTGCTCGGCGATGGACCAGA-3′;

*Ctsk* forward 5′-GATACTGGACACCCACTGGGA-3′ and reverse 5′-CATTCTCAGACACAATCCAC-3′;

*Mmp9*, forward 5′- GGAGCACGGCAACGGAGAAG-3′ and reverse 5′- CCTGGTCATAGTTGGCTGTGGTG-3′;

*Calcr*, forward 5′-ATTTTGCCACTGCCTTTCAG-3′ and reverse 5′-ATTTTCTCTGGGTGCGCTAA-3′;

NFATc1, forward 5′-TGTTCTTCCTCCCGATGTCT-3′ and reverse 5′-CCCGTTGCTTCCAGAAAATA-3′;

c-fos, forward 5′-TTGCTGATGCTCTTGACTGG-3′ and reverse 5′-GGATTTGACTGGAGGTCTGC-3′;

GAPDH, forward 5′-AAATGGTGAAGGTCGGTGTG-3′ and reverse 5′-TGAAGGGGTCGTTGATGG-3′.

### Animal treatments

The Animal Welfare Committee in Zhejiang University School of Medicine approved all animal care activities. Female C57BL/6 mice were randomly divided into four groups with 5 mice in each group at 10–12 weeks of age: (a) PBS, (b) rhEPO, (c) rhEPO + GW9962, and (d) rhEPO + ZOL. The rhEPO was administered intravenously at a dose of 5000 IU/kg three times per week for 2 months. The same volume of PBS, GW9962 (1 mg/kg), and ZOL (2 mg/kg,) was given to the control and rhEPO + ZOL group. rhEPO was obtained from Sunshine Pharmaceutical (Shenyang, China) for patient care.

### Micro-computed tomography analysis

After mice were sacrificed, the femora (1 per mouse) were soaked in ethanol. We used a micro-CT (Analyze 12.0, PerkinElmer) to scan the femurs. We scanned the entire length of the femur at a pixel size of 15 μm, and analyzed the results according to the manufacturer’s instructions. Region-of-interest (ROI) was defined from 0.225 mm (15 image slices) to 2.475 mm (165 image slices), with the growth plate slice defined as 0 mm. We performed an analysis of bone-related parameters in the ROI region. We used 3D models to enhance the interpretation of the images by reconstructing the data specifically from the ROI region. The software (Analyze 12.0, PerkinElmer) was used to analyze trabecular microstructural parameters, including bone mineral density (BMD), trabecular number (Tb. N), trabecular bone volume per tissue volume (BV/TV), trabecular thickness (Tb. Th) and trabecular separation (Tb. Sp).

### TRAP staining of tibias

After animal sacrifice, the tibias per mouse were fixed in 4% paraformaldehyde for 48 h. After the samples were decalcified in 0.5 M EDTA for 4 weeks, they were embedded in paraffin. Five-micrometers-thick sections of the tibias were performed the tartrate-resistant acid phosphatase (TRAP) staining using the standard protocol.

### ELISA Analysis and blood analysis

After the final dose of injection, blood was collected by enucleation of the eyeballs, and serum was collected by separation from the clotted blood for ELISA assay and blood analysis. 0.5 mL mouse whole blood was collected in a 2 mL centrifuge tube containing 2.5 µL of 2% heparin sodium. Then, blood parameters (hematocrit, RBCs and hemoglobin) were measured using a Sysmex automated blood cell counter (Sysmex XE-2100 and XE-5000). 1mL mouse whole blood was collected in a 2 mL centrifuge tube. Serum was collected from the supernatant of centrifuged mouse whole blood. Then, bone metabolism level was evaluated by measuring the concentration of serum C-terminal telopeptides of type I collagen (CTX-1). Serum CTX-1 levels were analyzed using a mouse CTX-1 EIA kit (Immunodiagnostic Systems) according to the manufacturer’s instructions.

### Statistical analysis

The data is presented as the mean ± standard deviation (SD). Statistical analysis was performed using SPSS 20.0 (SPSS Science Inc., Chicago, Illinois). Statistical significance was assessed using a one-way analysis of variance (ANOVA) analysis. A significant difference was considered as a P value < 0.05.

## Results

### EPOR is expressed in BMMs

**Fig. 1:**
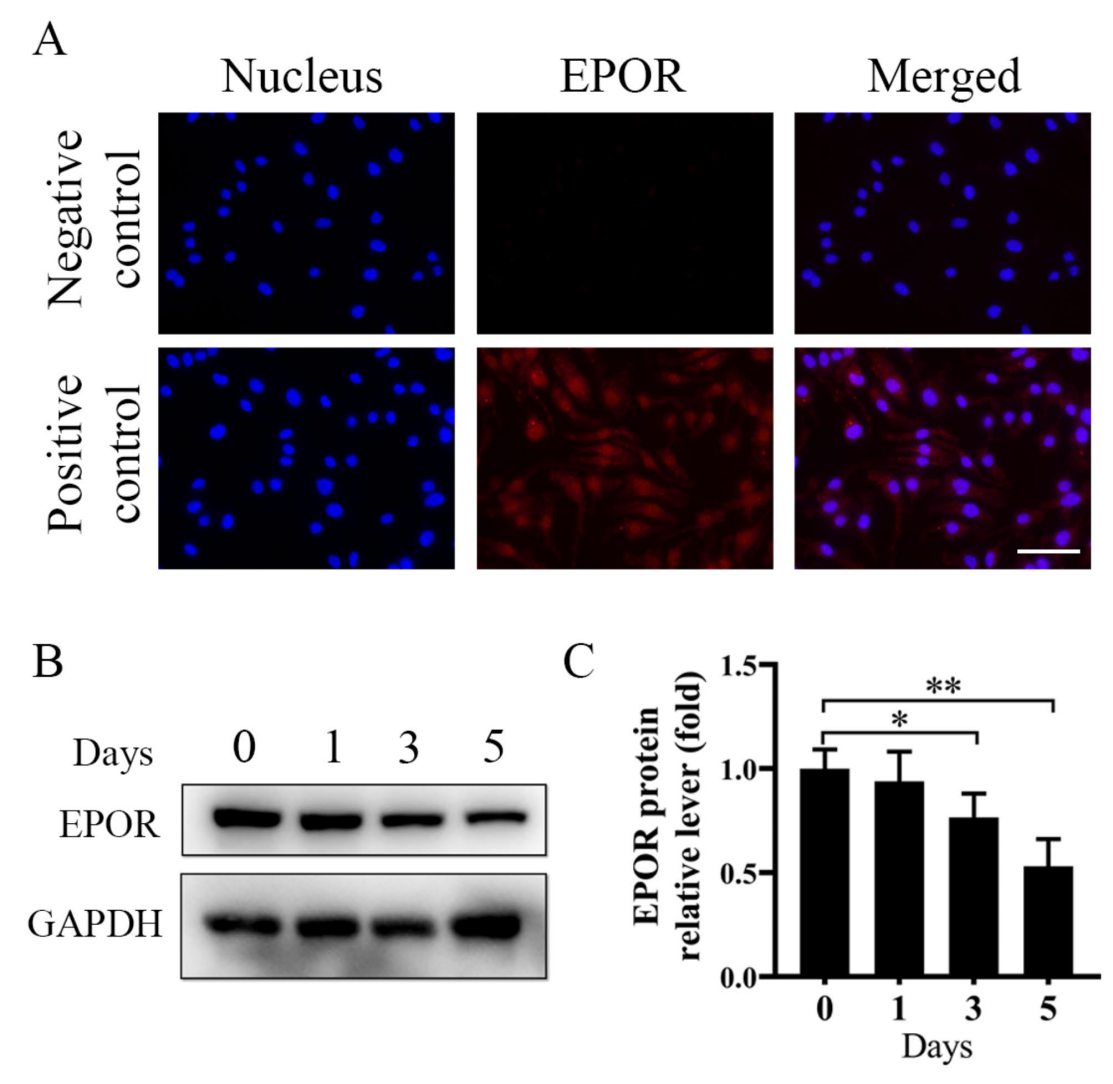
EPOR is expressed on BMMs. **(A)** Confocal analysis of EPOR (red) expression on BMMs. Scale bar = 100 μm. (**B**, **C**) Western blots showing the expression of EPOR on 0, 1, 3, and 5 days after osteoclastic induction, and its quantification; *n* = 3. Error bars are mean ± SD; *P < 0.05; **P < 0.01; NS, not significant

It is well known that EPO can induce hematopoiesis via EPOR expressed on erythroid progenitor cells. However, our results showed that EPOR was identified as being expressed on BMMs (Fig. 1A), which was consistent with the previous report (Lifshitz et al. 2010). Moreover, we observed a decrease in EPOR levels from the beginning of RANKL-induced osteoclastogenesis when M-CSF was present (Fig. 1B, C).

### EPO promotes RANKL-induced osteoclast differentiation

**Fig. 2:**
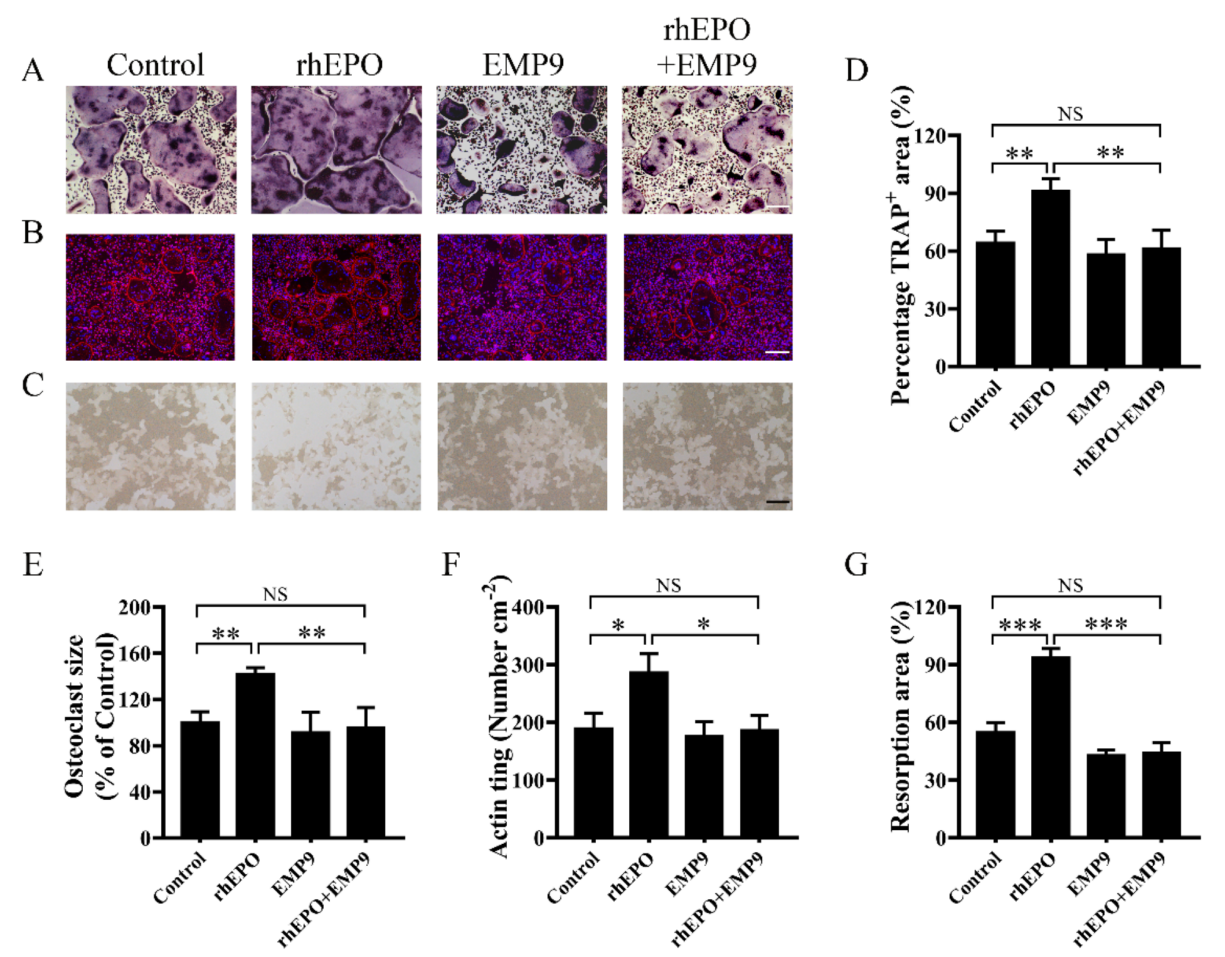
EPO enhances RANKL-induced osteoclast differentiation and bone resorption in vitro. **(A)** Representative TRAP staining images of BMMs treated with 30 ng/mL M-CSF and 50 ng/mL RANKL for four days in the presence of EPO and EMP9. Scale bar = 200 μm. **(B)** Representative images of osteoclasts with actin ring (red) and cell nucleus (blue); the BMMs were treated with 30 ng/mL M-CSF and 50 ng/mL RANKL for four days in the presence of EPO or/and EMP9. Scale bar = 200 μm. **(C)** Representative images of bone resorption pits on Corning Osteoassay 24-well plates. Scale bar = 200 μm. **(D)** The area of TRAP^+^ multinucleated cells (nuclei > 3) was quantified in each group. **(E)** The relative cell size of TRAP^+^ multinuclear cells (nuclei > 3) was quantified in each group. **(F)** EPO increased the number of multinucleated cells (nuclei > 3) with actin rings. **(G)** The relative resorption area was quantified by using ImageJ Software. Error bars are mean ± SD of triplicate experiments; *P < 0.05; **P < 0.01; ***P < 0.001; NS, not significant

Biologically, osteoclasts are commonly differentiated from BMMs under treatment with recombinant M-CSF and RANKL in vitro. The cells were treated with rhEPO, EMP9, or a combination during the differentiation of BMMs into osteoclasts. The results showed that rhEPO increased the area of TRAP^+^ osteoclast and the relative cell size of TRAP^+^ multinuclear osteoclast (nuclei > 3) after four days of incubation for cell differentiation as compared to the control (Fig. 2A, D, and E). Moreover, we found that EMP9 (an EPOR antagonist) (Luo et al. 2013) suppressed the EPO-enhanced osteoclast formation (Fig. 2A, D, and E; Figure S1). The formation of actin rings, a unique cytoskeletal structure found in mature osteoclasts that facilitates bone absorption (Geng et al. 2017), was also superior in rhEPO-treated cells compared to the control group, and EMP9 suppressed the EPO-enhanced actin ring formation (Fig. 2B, F). Furthermore, it was found to separately enhance the bone resorption activity of osteoclasts, which was also reversed by EMP9 (Fig. 2C, G). The data demonstrated that EPO/EPOR signaling in BMMs could enhance osteoclast differentiation and bone resorption in vitro.

### EPO up-regulated osteoclastogenic gene expression

**Fig. 3:**
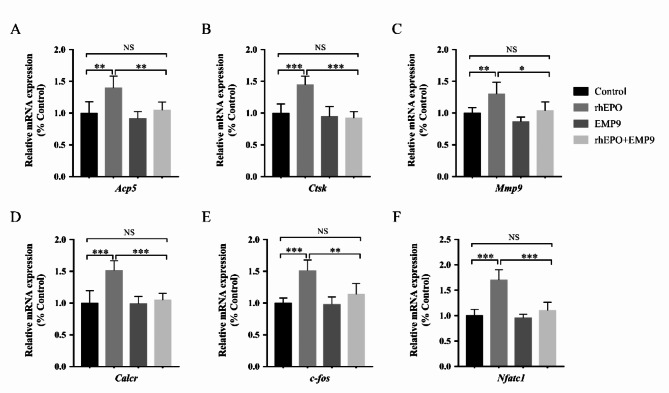
EPO up-regulated mRNA expression of RANKL-induced osteoclast-specific functional genes and transcription factors. EPO up-regulated osteoclast-specific functional genes **(A)** *Acp5*, **(B)** *Ctsk*, **(C)** *Mmp9*, **(D)** *Calcr*, and transcription factors **(E)** *c-fos*, **(F)** *Nfatc1* expression. Error bars are mean ± SD of triplicate experiments; *P < 0.05; **P < 0.01; ***P < 0.001; NS, not significant

Mature osteoclasts express several specific genes that are vital for extracellular matrix degradation and bone resorption (Jacome-Galarza et al. 2019). Their gene products include TRAP, cathepsin K, matrix metalloproteinase-9, and calcitonin receptor (McDonald et al. 2021). Our results showed that EPO up-regulated the expression of these genes by approximately 120–150%, which was effectively inhibited by the EPOR antagonist EMP9 (Fig. 3A-D). RANKL binding to its receptor RANK initiates a complex signaling cascade that regulates lineage commitment and osteoclast differentiation (Shinohara et al. 2008). It was reported that c-fos plays a crucial role in mediating osteoclast differentiation; mice that lack c-fos developed osteosclerosis due to impaired osteoclast differentiation (Grigoriadis et al. 1994). RANKL induces c-fos expression, which is required to induce the nuclear factor of activated T cells and cytoplasmic calcineurin-dependent-1 (NFATc1), a central transcription factor that governs osteoclast differentiation (Matsuo et al. 2000). Notably, EPO treatment significantly up-regulated the expression of c-fos and NFATc1, while this effect was abrogated by EMP9 (Fig. 3E, F).

### EPO promotes PPARγ expression during osteoclast differentiation

**Fig. 4:**
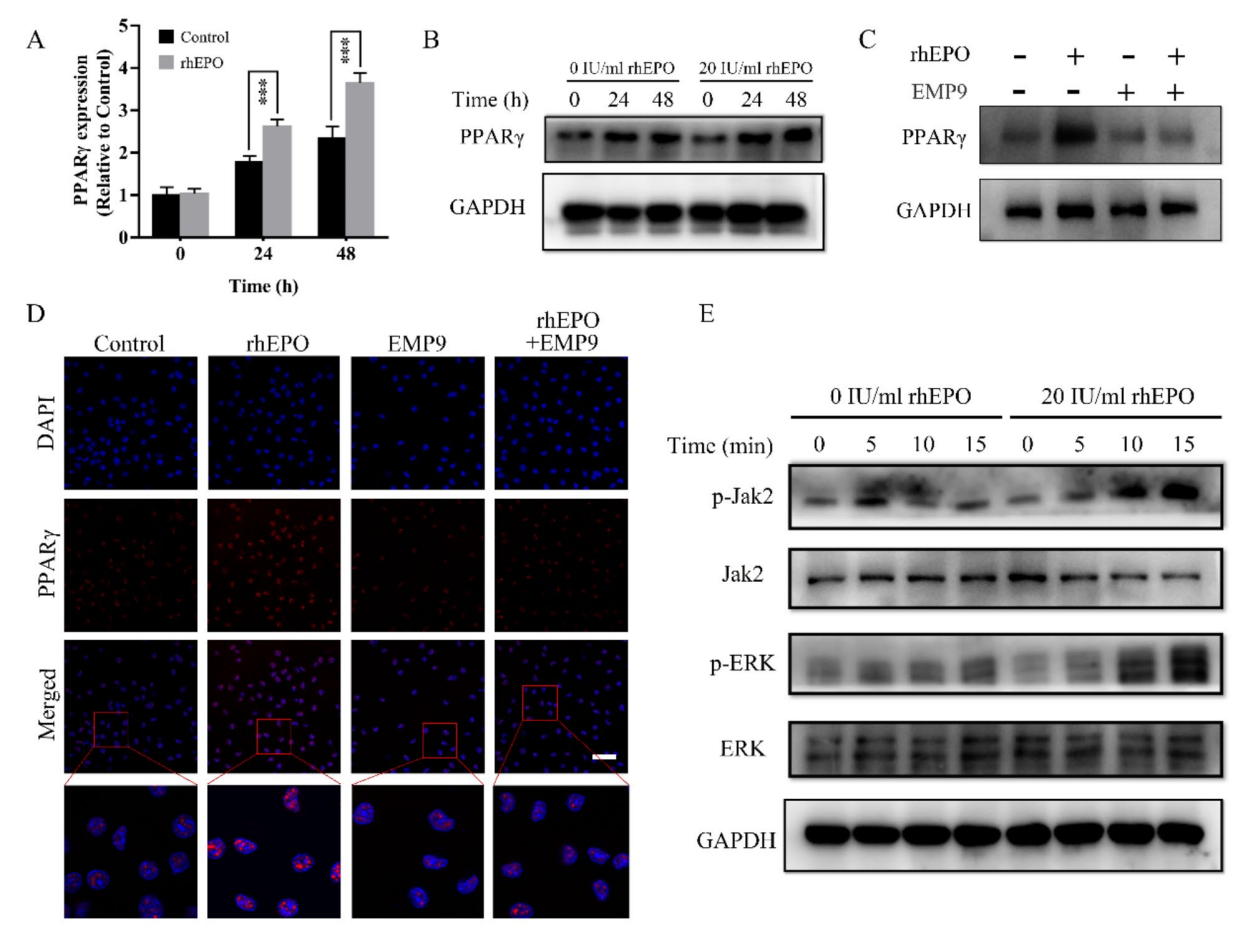
EPO induced PPARγ expression during osteoclast differentiation. **(A)** EPO up-regulated the transcription factor PPARγ mRNA expression at indicated time points. **(B)** EPO up-regulated the transcription factor PPARγ protein expression at indicated time points. **(C)** BMMs were incubated with 30 ng/mL M-CSF and 50 ng/mL RANKL in the presence of EPO or/and EMP9 for 24 h, and the PPARγ protein expression was measured using Western blot. **(D)** The PPARγ protein expression was measured using immunofluorescence after different treatments. **(E)** The phosphorylated (p-) and total Jak2 and ERK protein expression were measured after different treatments at indicated time points. Error bars are means ± SD, *n* = 3; *P < 0.05; **P < 0.01; ***P < 0.001; NS, no significant

We investigated the mechanisms involved in EPO/EPOR-mediated osteoclast differentiation. EPO binding to its receptor EPOR activates the STAT5, PI3K-AKT, and MEK-ERK, STAT5 pathways, inducing Jak2 phosphorylation (Nairz et al. 2012). Among these cascades, a previous study confirmed that the activation of ERK increased the PPARγ expression in macrophages (Luo et al. 2016). Moreover, PPARγ directly regulated the expression of c-fos to promote osteoclastogenesis (Wan et al. 2007). Therefore, we hypothesized that EPO might promote osteoclast differentiation via the Jak2-ERK-PPARγ pathway. Since EPO up-regulated the expression of transcription factors c-fos (Fig. 3E), we examined the expression of PPARγ in response to EPO treatment. The results showed that EPO increased the mRNA and protein amounts of PPARγ in a time-dependent manner during osteoclastogenesis (Fig. 4A, B). Moreover, the elevated expression of PPARγ protein by EPO could be reversed by EMP9 (Fig. 4C, D). Furthermore, the activation of Jak2 and ERK was induced by EPO in a time-dependent manner, while the total protein amounts of Jak2 and ERK were not influenced by EPO (Fig. 4E). The Jak2 inhibitor (AG490) attenuated EPO-induced PPARg expression and the activation of Jak2 and ERK in BMMs (Figure S2B). The ERK inhibitor (PD98059) decreased EPO-induced PPARγ expression and ERK activation but not Jak2 in BMMS (Figure S2B). The PPARγ siRNA suppressed the PPARg expression but did not affect Jak2 and ERK in BMMs (Figure S2 A, B). The present data indicated that EPO promoted osteoclast differentiation via the Jak2-ERK-PPARγ cascade.

### EPO promoted RANKL-induced osteoclast differentiation through PPARγ

**Fig. 5:**
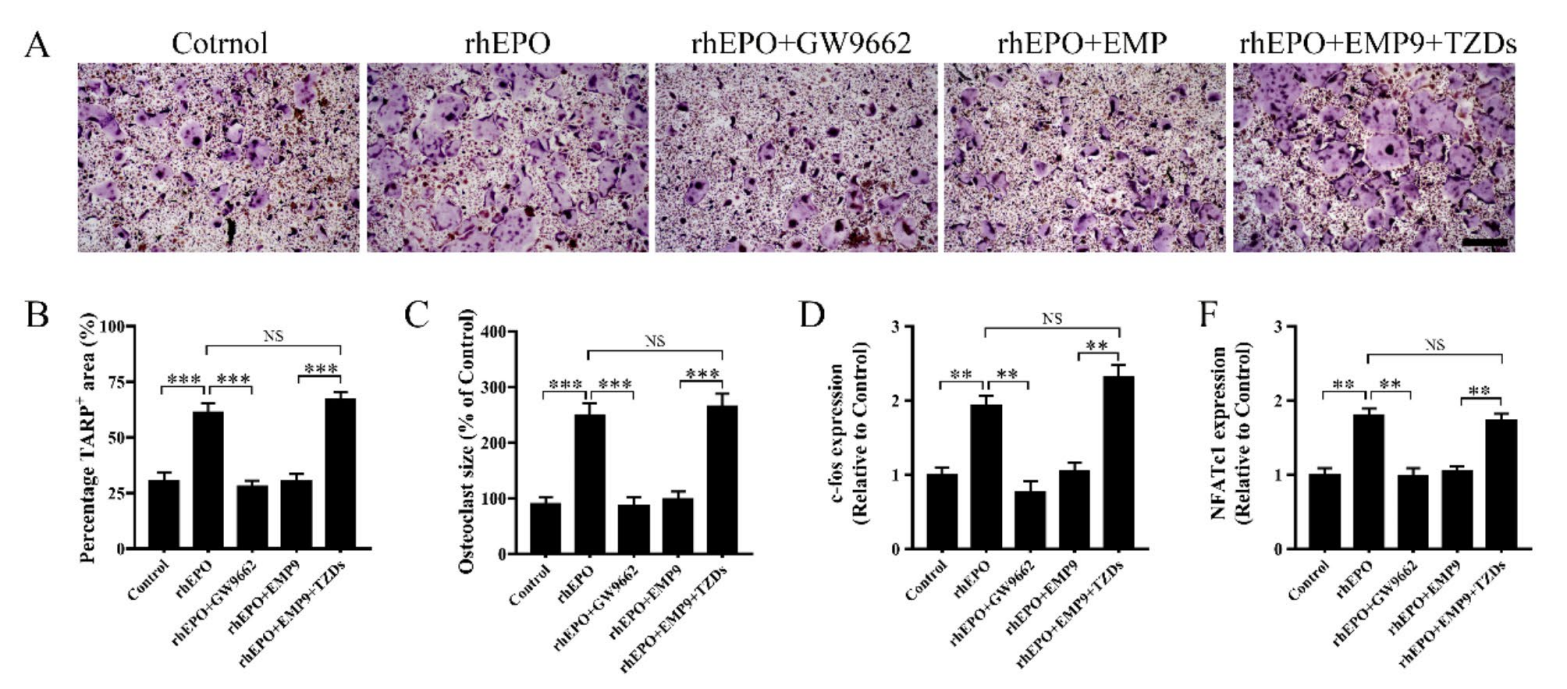
EPO enhanced osteoclast differentiation via inducing PPARγ. **(A)** Representative TRAP staining images of BMMs treated with 30 ng/mL M-CSF and 50 ng/mL RANKL for 4 days in the presence of EPO/EPO + GW9662/EPO + EMP9/EPO + EMP9 + TZDs. Scale bar = 200 μm. **(B)** The area of TRAP^+^ multinucleated cells (nuclei > 3) was quantified in each group. **(C)** The relative cell size of TRAP-positive multinuclear cells (nuclei > 3) was quantified in each group. (**D**, **E**) RT-qPCR was used to detect the mRNA expression of transcription factors **(D)** *c-fos* and **(F)***Nfatc1*. Error bars are mean ± SD of triplicate experiments; **P < 0.01; ***P < 0.001; NS, no significant

PPARγ is important for osteoclastogenesis. Loss of function by targeted PPARγ deletion impairs osteoclast differentiation and bone resorption, resulting in osteopetrosis (Wan et al. 2007). GW9662 (a PPARγ antagonist) significantly suppressed EPO-promoted osteoclast differentiation. Moreover, thiazolidinediones (TZDs, a PPARγ agonist) could reverse the EMP9-mediated decrease in osteoclast differentiation (Fig. 5A-C). Furthermore, GW9662 suppressed EPO-induced mRNA expression of transcription factors c-fos and NAFATc1, and TZDs could reverse EMP9-mediated down-regulation of c-fos and NAFATc1 expression (Fig. 5D, F). These results indicated that EPO promotes osteoclast differentiation through PPARγ.

### Epo induced trabecular bone loss in adult female mice

**Fig. 6:**
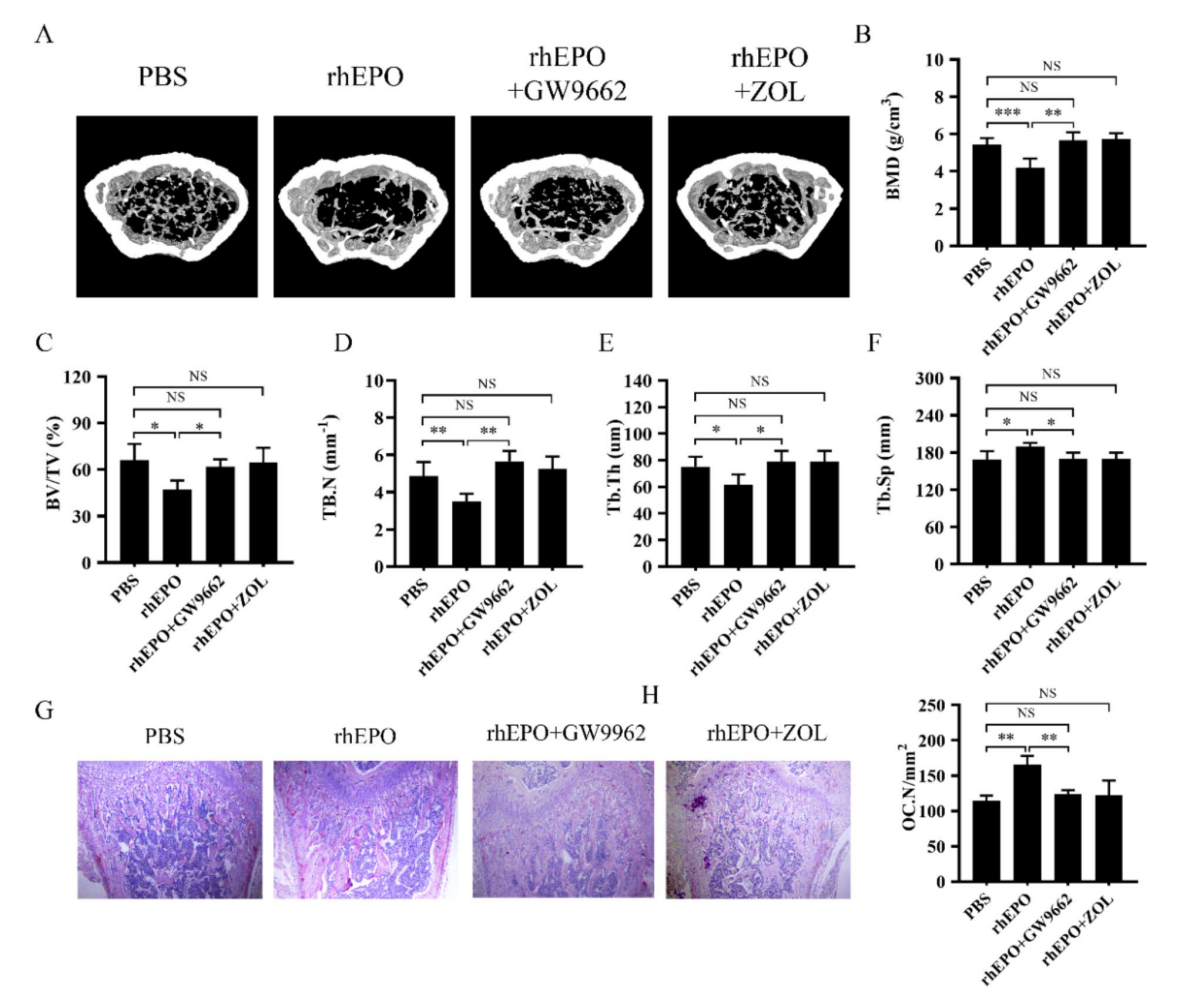
EPO increased bone loss in vivo. **(A)** Representative micro-CT images of mouse distal femur. (**B**–**F**) Quantitative micro-CT analysis of BMD, BV/TV, Tb.N, Tb. Th and Tb.Sp. **(G)** Light micrographs of TRAP-staining of decalcified bones. Scale bar = 200 μm. **(H)** Quantification of osteoclast number (Oc.N) in G. Error bars are means ± SD, *n* = 5; *P < 0.05; **P < 0.01; ***P < 0.001; NS, no significant

As EPO promotes osteoclast differentiation, we examined the effects of EPO on bone loss. We used micro-CT to quantify the bone density by analyzing various bone parameters. As previously reported, EPO significantly induced bone loss (Fig. 6A) (Hiram-Bab et al. 2015). Moreover, EPO significantly decreased bone mineral density (BMD), volume/total volume (BV/TV), trabecular number (Tb.N), and trabecular thickness (Tb.Th) (Fig. 6B-E), and increased trabecular separation (Tb.Sp) (Fig. 6F). After analyzing micro CT parameters, the BMD, BV/TV, Tb.N, Tb.Th, and Tb.Sp were all restored to the level of the control group with the injection of GW9662 and zoledronate (ZOL) (Fig. 6A-F). ZOL, the third-generation drug of bisphosphonates with strong bone affinity, has been employed to treat osteoporosis by inhibiting osteoclastogenesis. Then we used histological TRAP staining to label osteoclasts in the bone trabecula region. EPO increases TRAP-positive staining in the trabecula region compared with the PBS group. However, GW9662 and ZOL reversed the effect of EPO on TRAP-positive staining (Fig. 6G, H). Finally, we evaluated bone metabolism level by measuring serum bone resorption marker C-terminal telopeptides of type I collagen (CTX-1) and bone formation marker procollagen type I N-terminal propeptide (PINP). As speculated, EPO significantly in-creased systemic bone resorption, which was also reversed by GW9662 and ZOL (Figure S3). EPO and ZOL did not affect bone formation levels. Moreover, EPO significantly increased the number of red blood cells, hemoglobin lever and hematocrit compared with PBS group. EPO in combination with GW9962 and EPO with ZOL group exhibited red blood cell parameters similar to those of EPO group. (Figure S4).

## Discussion

Researchers showed that the administration of high doses of EPO to adult mice induces bone loss (Rauner et al. 2016; Suresh et al. 2020a, b, c). EPO can induce bone loss by increasing bone resorption by osteoclasts and decreasing bone formation by osteoblasts. Notably, excessive or overactive osteoclasts are The primary reason for bone loss in disorders like osteoporosis and Paget’s disease is the presence of excessive or overactive osteoclast (Kim et al. 2021), Therefore, a viable strategy for treating these diseases is to focus on inhibiting the differentiation and activity of osteoclasts. This study demonstrated that EPO promoted osteoclastogenesis and bone resorption in vitro and in vivo. The molecular mechanism by which EPO/EPOR stimulates osteoclastogenesis is the regulation of downstream PPARγ.

Suresh et al. previously reported that EPOR is expressed in nonerythroid cells (Suresh et al. 2019) Our results showed that EPOR is also expressed in BMMs (Fig. 1A). However, we observed decreased EPOR expression from initiating RANKL-induced osteoclastogenesis in the presence of M-CSF (Fig. 1B, C), suggesting that RANKL signaling may down-regulate EPOR during osteoclastogenesis. Functional EPOR is expressed by several non-erythroid cells, indicating that EPO may have a broad regulatory role beyond erythropoiesis (Suresh et al. 2020a, b, c). In this study, we specifically evaluated the effect of EPO/EPOR signaling on osteoclast differentiation and observed that EPO increased both osteoclast formation and bone resorption ability. These findings are consistent with the study by Hiram-Bab et al., which demonstrated that even a low dose of EPO stimulated osteoclastogenesis (Hiram-Bab et al. 2017; Kim et al. 2012). However, Singbrant et al. reported both EPO and erythroblasts did not stimulate osteoclast differentiation in cocultures of BMMs and calvarial osteoblasts (Singbrant et al. 2011). We speculated that this may be because EPO inhibits the formation of osteoblast, thus weakening the coupling between osteoblast and osteoclast. Other studies supported that EPO can promote osteoclast differentiation, consistent with our findings (Shiozawa et al. 2010; Li et al. 2015).

However, the mechanism by which EPO promotes osteoclast differentiation is unclear. Although EPO is well-known for its ability to promote erythropoiesis, recent studies have found that EPO has extrahematopoietic properties via EPORs expressed in nonhematopoietic tissues. For instance, rhEPO activates the PI3K/Akt to upregulate PPARγ, enhance the cellular antioxidant capacity, and protect neurons in rats subjected to oxidative stress (Wang et al. 2022). Furthermore, Ge et al. found that EPO/EPOR signaling activates the hepatic AKT pathway by increasing PPARγ expression and activity, which improvements in hepatic insulin resistance (Ge et al. 2015).

Biologically, osteoclasts are derived from the monocyte/macrophage lineage in response to RANKL (Bae et al. 2023). RANKL binding to RANK sequentially activates the downstream signaling pathways, ultimately inducing the expression and activation of c-fos and NFATc1, with two key transcription factors that drive osteoclast differentiation (Murata et al. 2022). In addition, EPO binding to its receptor EPOR activates the phosphorylation of Jak2 to activate MEK-ERK. This activation of ERK then increases the PPARγ expression in macrophages, which aids in the clearance of dying cells (Luo et al. 2016). Our results showed that EPO activated the phosphorylation of Jak2 and ERK in a time-dependent manner. Furthermore, EPO increased the expression of PPARγ in the downstream of JAK-ERK during osteoclastogenesis (Fig. 4G, F). Previous studies showed that PPARγ directly regulated the expression of c-fos to promote osteoclastogenesis (Wan et al. 2007; Wei et al. 2010). As c-fos is a crucial mediator in osteoclast formation, required for the induction of NFATc1 nuclear factor, mice lacking c-fos develop osteosclerosis due to the obstruction of osteoclast differentiation (Wada et al. 2006). Our results showed that EPO up-regulated the expression of transcription factors c-fos and NFATc1. Furthermore, GW9662 significantly suppressed EPO-promoted osteoclast differentiation by inhibiting the expression of transcription factors c-fos. Therefore, it is apparent that EPO functions through PPARγ to promote osteoclast differentiation.

Finally, we showed that exposure to EPO in adult mice resulted in bone loss, which was found to be associated with EPO-stimulated osteoclastogenesis. However, bone loss induced by EPO was reversed by ZOL. This finding is clinically significant as EPO is frequently used to treat anemia in postoperative patients with intertrochanteric fractures of the femur. As EPO treatment leads to bone loss, these patients may benefit from antiosteoporosis drugs such as denosumab and ZOL.

## Conclusion

**Fig. 7:**
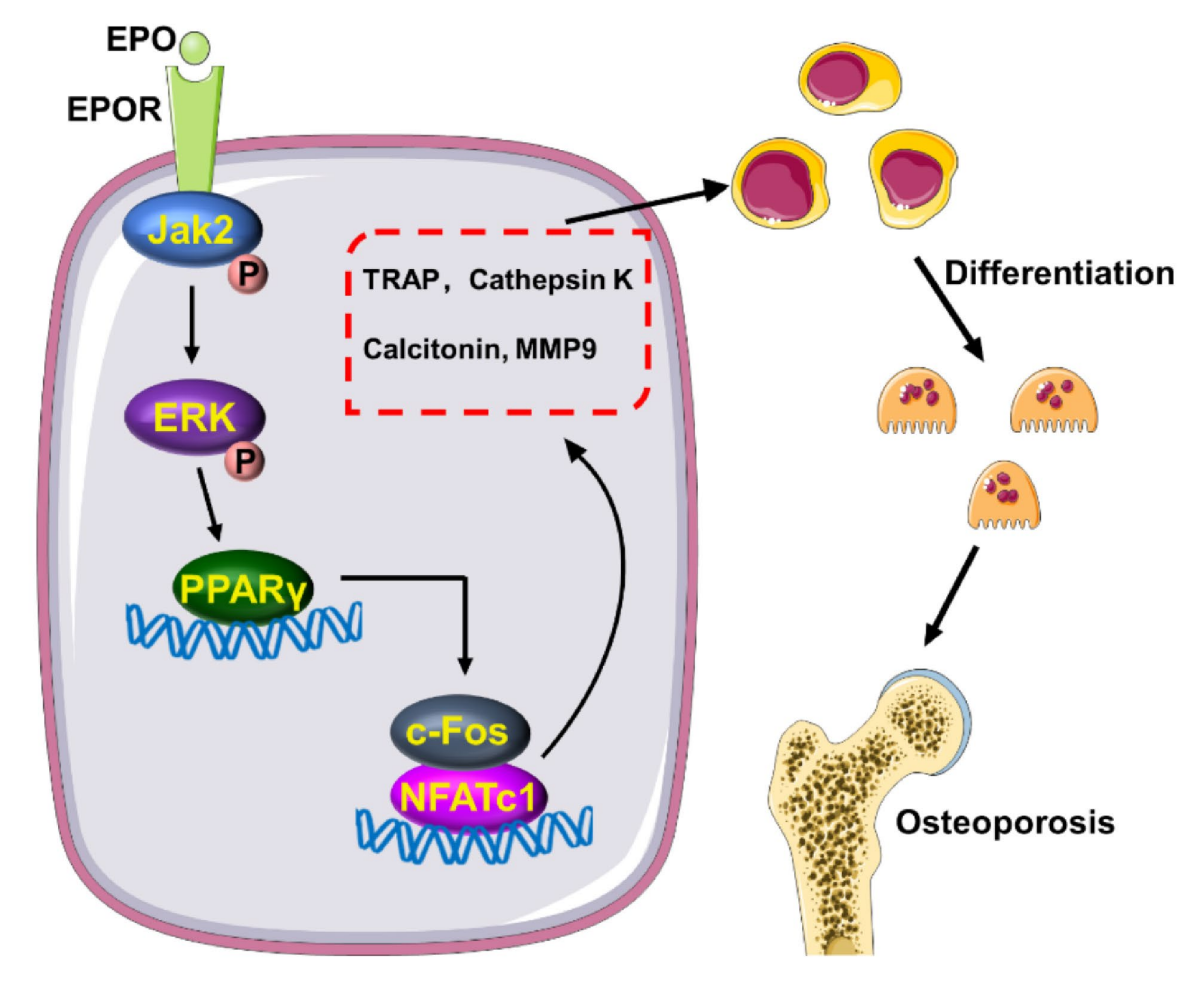
EPO binding to its receptor EPOR activates the Jak2/ERK signal pathway, increasing PPARγ expression. Then, PPARγ directly regulated the expression of c-fos to promote osteoclast differentiation and enhanced osteoclastic bone resorption

This study confirmed that EPO promoted RANKL-induced osteoclast differentiation and enhanced osteoclastic bone resorption. The underlying mechanism is related to the activation of the Jak2/ERK signal pathway, leading to an increased PPARγ expression (Fig. 7). Clinically, the risk of bone loss should be considered when using EPO to treat anemia.

## Electronic supplementary material

Below is the link to the electronic supplementary material.

Supplementary Material 1

Supplementary Material 2

## Data Availability

The original contributions presented in the study are included in the article; further inquiries can be directed to the corresponding authors.
