# Supplementary material for: Erythropoietin regulates osteoclast formation via up-regulating PPARγ expression
Source: Mol Med. 2024 Sep 15;30:151. doi: 10.1186/s10020-024-00931-7 (PMC11403934; doi:10.1186/s10020-024-00931-7)
Supplement: Supplementary file 1 — Supplementary Material 1 [file 10020_2024_931_MOESM1_ESM.docx]

**Erythropoietin regulates osteoclast formation via up-regulating PPARγ expression**

Xiao Liu^1^, Mengxue Zhou^2^, Yifan Wu^1^, Xiang Gao^1^, Lei Zhai^3^, Liang Liu^1,*^, Huan Geng^1,*^

*^1^ Department of Orthopedics, The Second Affiliated Hospital, School of Medicine, Zhejiang University, Hangzhou 310058, China*

*^2^ Key Laboratory of Tea Biology and Resource Utilization of Ministry of Agriculture, Tea Research Institute, Chinese Academy of Agricultural Sciences, Hangzhou 310008, China*

*^3^ Meiao Dingcheng Clinic Limited Company, Tianjin, 300000, China.*

*[*] Corresponding authors:*

*Dr. G. Huan (genghuan1989@zju.edu.cn), Department of Orthopedics, The Second Affiliated Hospital, School of Medicine, Zhejiang University, Hangzhou 310058, China;*

*Mr. L. Liu (llzju2014@163.com), Department of Orthopedics, The Second Affiliated Hospital, School of Medicine, Zhejiang University, Hangzhou 310058, China.*


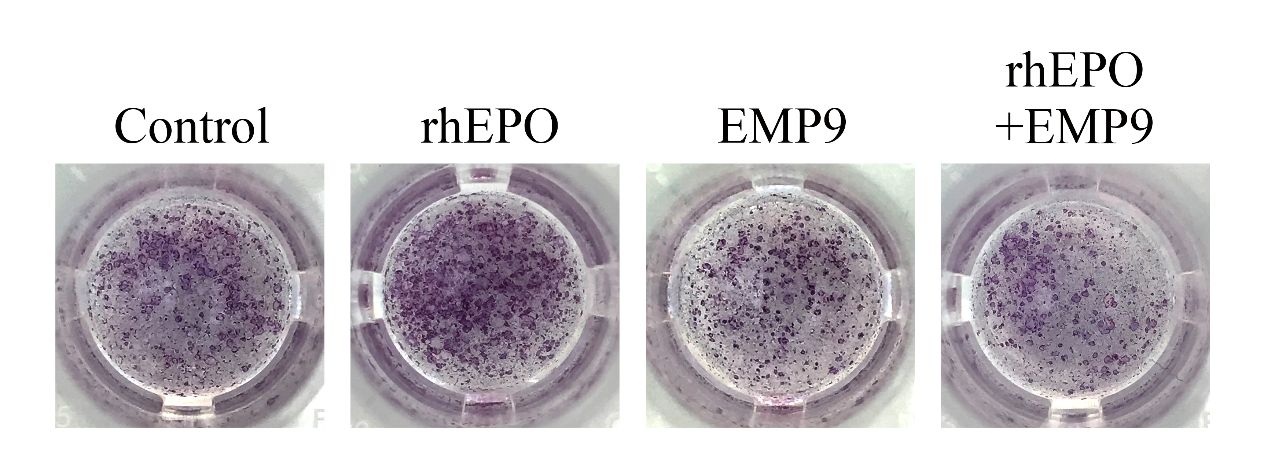


**Figure S1.** TRAP staining images of BMMs induced with 30 ng/mL M-CSF and 50 ng/mL RANKL for 4 days in the presence of EPO or/and EMP9. EPO enhances RANKL-induced osteoclast differentiation and bone resorption in vitro.


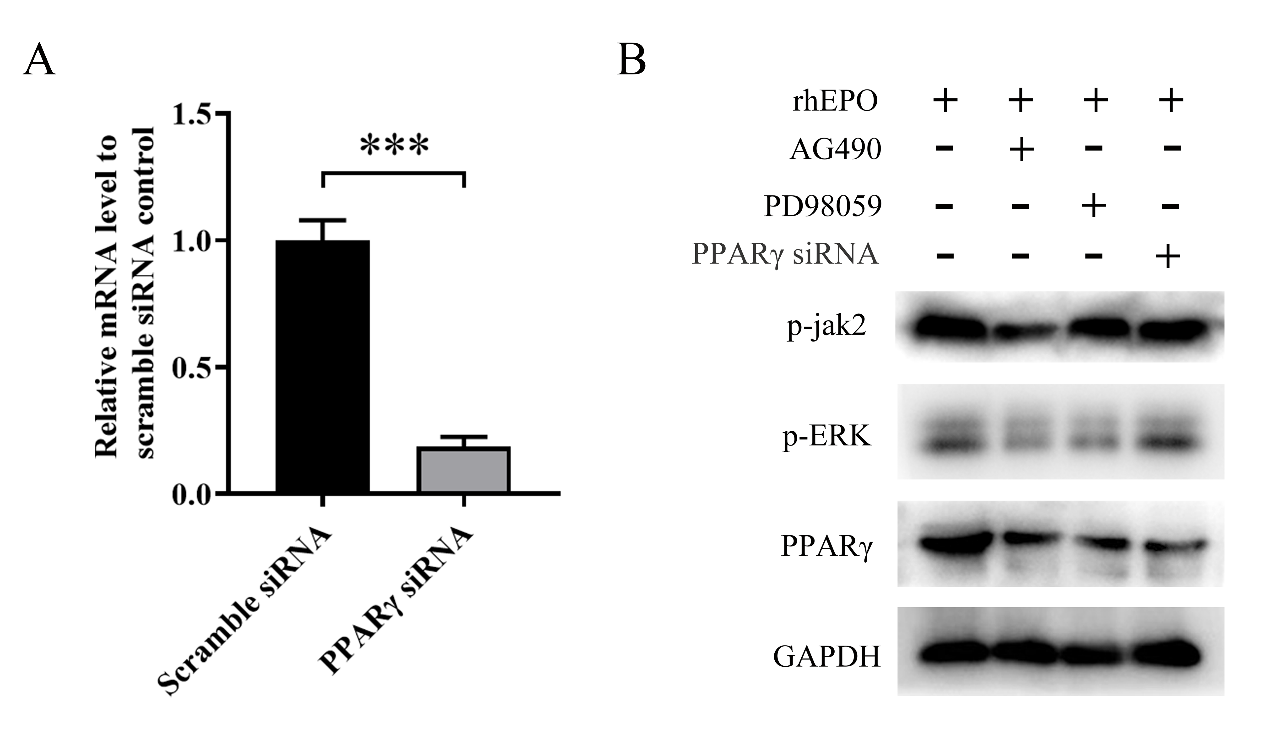


**Figure S2.** (A) PPARγ-specific siRNAs reduced PPARγ in BMMs. (B) BMMs were incubated with rhEPO, together with AG490 (Jak2 inhibitor), PD98059 (ERK inhibitor) or PPARγ siRNA for 6 h, and protein expression of related molecules were detected.


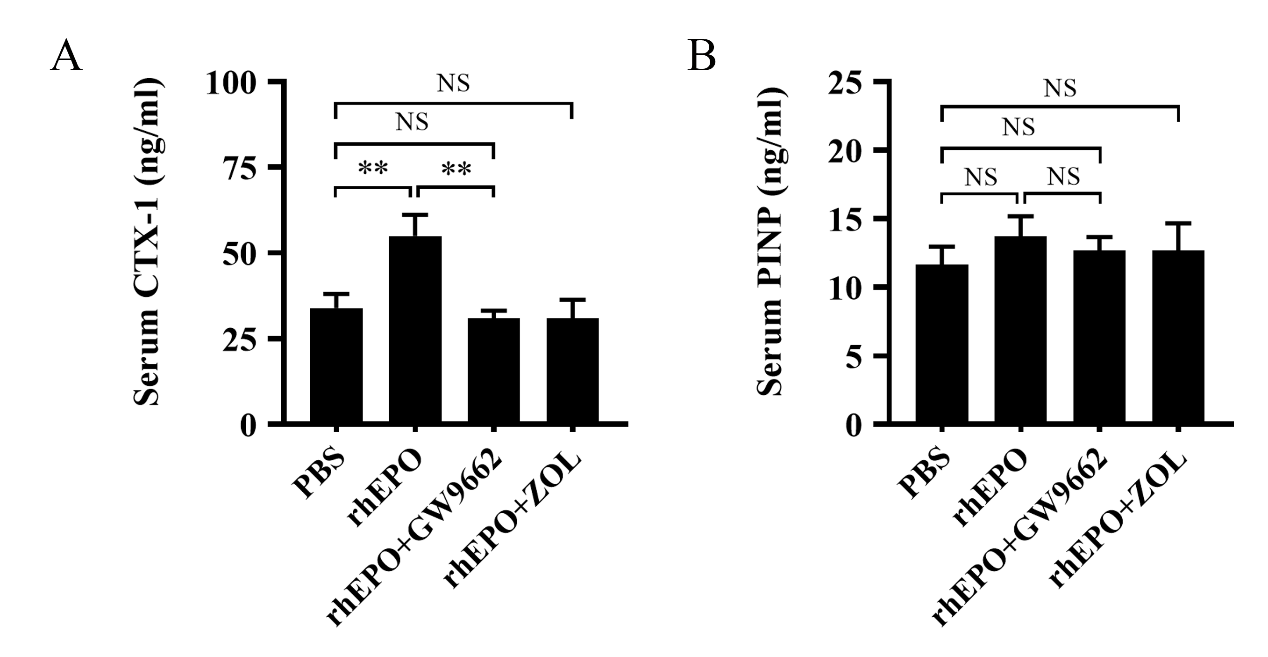


**Figure S3.** (A) Serum CTX-1 level was assayed by ELISA analysis. (B) Serum PINP level was assayed by ELISA analysis. Error bars are means ± SD, n = 5; **P* < 0.05; ***P* < 0.01; ****P* < 0.001; NS, no significant.


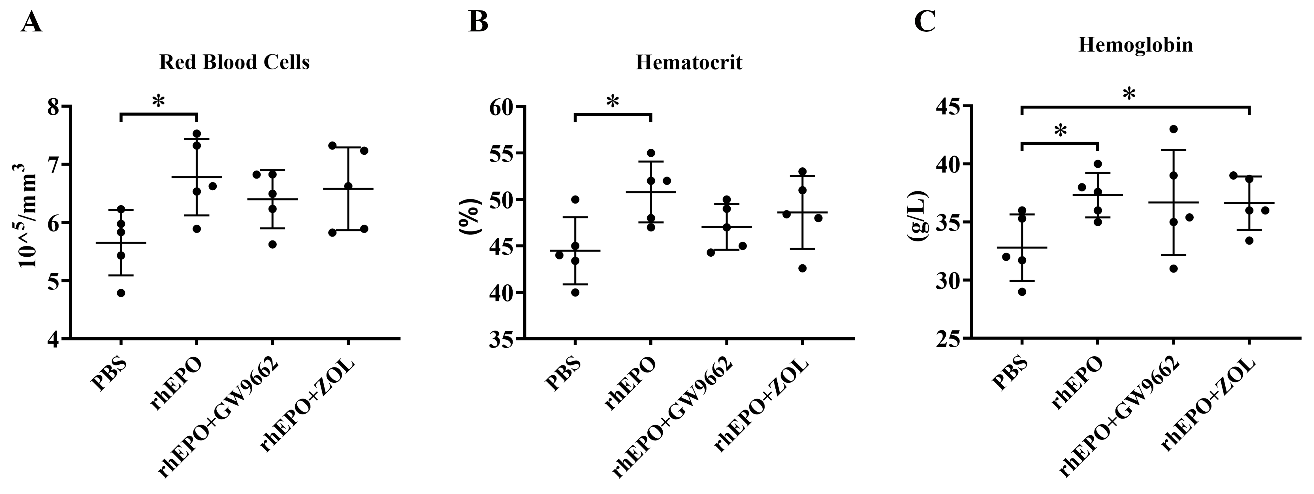


**Figure S4.** Blood parameters (A) red blood cells, (B) hematocrit and (C) hemoglobin were measured using a Sysmex automated blood cell counter. Error bars are means ± SD, n = 5; **P* < 0.05.
