# Supplementary material for: Erythropoietin regulates osteoclast formation via up-regulating PPARγ expression
Source: Mol Med. 2024 Sep 15;30:151. doi: 10.1186/s10020-024-00931-7 (PMC11403934; doi:10.1186/s10020-024-00931-7)
Supplement: Supplementary file 2 — Supplementary Material 2 [file 10020_2024_931_MOESM2_ESM.docx]

**Response to the Reviews**

**Reviewer #1:**

The authors follow previous publications that erythropoietin (EPO) adversely affects bone health and report here that EPO promoted osteoclast formation by upregulation of PPARg resulting in bone resorption in vitro, and that female mice treated with EPO for two months showed increased osteoclast numbers and decreased bone mass.

1.	Define "BMM." The definition of BMM was deleted in the current version.

**Response:** Thanks for your professional suggestions. BMMs is an abbreviation for bone marrow macrophages and we defined BMMs in the revised manuscript (Section 2.1 line 20):

*Mature osteoclasts were generated from bone marrow macrophages (BMMs) as described previously[10].*

2. Provide the anticoagulant that was used to collect blood for analysis of blood parameters.

**Response:** Thanks to the reviewer for this careful comment. For analysis of blood parameters, 0.5 mL mouse whole blood was collected in a 2 mL centrifuge tube containing 2.5 μL of 2% heparin sodium. Based on your suggestion, we have modified the method in Section 2.10:

*0.5 mL mouse whole blood was collected in a 2 mL centrifuge tube containing 2.5 μL of 2% heparin sodium. Then, blood parameters (hematocrit, RBCs and hemoglobin) were* *measured using a Sysmex automated blood cell counter (Sysmex XE-2100 and XE-5000). 1mL mouse whole blood was collected in a 2 mL centrifuge tube. Serum was collected from the supernatant of centrifuged mouse whole blood. Then, bone metabolism level was evaluated by measuring the concentration of serum C-terminal telopeptides of type I collagen (CTX-1).*

3. Authors should provide a better definition of ZOL such as ZOL belongs to the bisphosphate class of drugs.

**Response:** Thanks for your professional suggestions. According to the reviewer's suggestion, we have a better explanation of ZOL:

*After analysis of micro CT parameters, the BMD, BV/TV, Tb.N, Tb.Th, and Tb.Sp were all restored to the level of the control group with the injection of GW9662 and zoledronate (ZOL) (Figure 6 A-F). ZOL, the third-generation drug of bisphosphonates with strong bone affinity, has been employed to treat osteoporosis by inhibiting osteoclastogenesis.*

4. Line 15, page 19: "Furthermore, Ge et al. found that …aids in the clearance of dying cell[19]." The text requires clarification. Reference 30 focuses on EPO activity in hepatocytes and the text implies that this is related to PPARg expression in macrophages. However, reference 30 is about hepatocyte response to EPO and does not discuss EPO modification of PPARg expression in macrophages.

**Response:** We would like to thank the reviewer for pointing out this issue. Based on your suggestion, we have revised text to eliminate the reader's misunderstanding:

*However, the mechanism by which EPO promotes osteoclast differentiation is unclear.* *Although EPO is well-known for its ability to promote erythropoiesis, recent studies have found that EPO has extrahematopoietic properties via EPORs expressed in nonhematopoietic tissues. For instance, rhEPO activates the PI3K/Akt to upregulate PPARγ, enhance the cellular antioxidant capacity, and protect neurons in rats subjected to oxidative stress[29]. Furthermore, Ge et al. found that EPO/EPOR signaling activates the hepatic AKT pathway by increasing PPARγ expression and activity, which improvements in hepatic insulin resistance [30].*

*Biologically, osteoclasts are derived from the monocyte/macrophage lineage in response to RANKL[31]. RANKL binding to RANK sequentially activates the downstream signaling pathways, ultimately inducing the expression and activation of c-fos and NFATc1, with two key transcription factors that drive osteoclast differentiation[8]. In addition, EPO binding to its receptor EPOR activates the phosphorylation of Jak2 to activate MEK-ERK. This activation of ERK then increases the PPARγ expression in macrophages, which aids in the clearance of dying cells [19].*

5. References should be carefully reviewed and corrected.

Ref. 29 is not correct. Authors are not Huqing W, Ming C, Tao Z, et al. Correction required: Wang H, Chen M, Zhang T, Gao Z, Gong Y, Yu X, Wu H. Recombinant human erythropoietin upregulates PPARγ through the PI3K/Akt pathway to protect neurons in rats subjected to oxidative stress. Eur J Neurosci. 2022 Aug;56(3):4045-4059. doi: 10.1111/ejn.15735. Epub 2022 Jun 23. PMID: 35678781.

Ref 30 is not correct. Authors are not Zhijuan G, Pengzi Z, Ting H et al. Correction required: Ge, Z., Zhang, P., Hong, T. et al. Erythropoietin alleviates hepatic insulin resistance via PPARγ-dependent AKT activation. Sci Rep 5, 17878 (2016).

**Response:** We sincerely appreciate the reviewers for pointing out our errors. We have corrected this error in the manuscript:

*29.*	*Wang H, Chen M, Zhang T, et al:* ***Recombinant human erythropoietin upregulates PPARγ through the PI3K/Akt pathway to protect neurons in rats subjected to oxidative stress.*** *Eur J Neurosci 2022, 56:4045-4059.*

*30. Ge, Z., Zhang, P., Hong, T, et al:* ***Erythropoietin alleviates hepatic insulin resistance via PPARγ-dependent AKT activation****. Sci Rep 2015, 5:17878.*
